# Addressing smokeless tobacco use and building research capacity in South Asia (ASTRA)

**DOI:** 10.7189/jogh.10.010327

**Published:** 2020-06

**Authors:** Anne Readshaw, Ravi Mehrotra, Masuma Mishu, Zohaib Khan, Faraz Siddiqui, Kathryn Coyle, Kamran Siddiqi, on behalf of the ASTRA Global Health Research Group

**Affiliations:** 1 Department of Health Sciences, University of York, York, UK; 2 India Cancer Research Consortium, Indian Council of Medical Research – Department of Health Research, New Delhi, India; 3 Office of Research, Innovation and Commercialization, Khyber Medical University, Peshawar, Pakistan; 4 Department of Clinical Sciences, Brunel University, London, UK

ASTRA (Addressing Smokeless Tobacco Use and Building Research Capacity in South Asia) is a new Global Health Research Group funded by the UK’s National Institute for Health Research (NIHR). The aim of ASTRA is to reduce the burden of disease caused by the use of smokeless tobacco in South Asian countries.

The ASTRA consortium comprises five UK Universities and six partner organizations from Bangladesh, India and Pakistan. Coordinated by the University of York, ASTRA’s UK partners include the University of Edinburgh, King’s College London, Brunel University London and the University of Warwick. Our South Asian partners include ARK Foundation (Bangladesh), Aga Khan University (Pakistan), Khyber Medical University (Pakistan), Maulana Azad Medical College (India), the National Institute of Cancer Prevention and Research (NICPR, India) and HRIDAY (Health Related Information Dissemination Amongst Youth), India. We are supported by key stakeholders, including the World Health Organization (WHO) and the International Union Against Tuberculosis and Lung Disease (The UNION). The governance, activities and progress of ASTRA are overseen by an International Advisory Board (IAB), comprising members from the National Cancer Institute (USA), the American Cancer Society, Brown University, NICPR and Bangladesh’s National Heart Foundation Hospital and Research Institute.

## SMOKELESS TOBACCO AND THE WHO-FRAMEWORK CONVENTION ON TOBACCO CONTROL

Smokeless tobacco (ST) is a broad term describing a diverse range of tobacco-containing products that are consumed by chewing, keeping in the mouth or sniffing, rather than smoking. Eighty per cent of the world’s 300 million consumers of ST live in South Asia, where ST use is integrated into mainstream culture [1]. Common ST products favoured in South Asian countries include *naswar*, *gutkha*, *zarda, khaini* and *tambaku paan;* all of which contain nicotine and are highly addictive. Although recipes and ingredients vary widely, many contain high levels of heavy metals and carcinogens [1]. As a result, ST use leads to various types of head and neck cancers and has been implicated in cardiovascular diseases and poor birth outcomes [2-4]. In 2010, ST use was estimated to cause over 250 000 deaths and the loss of more than 6 million disability adjusted life years (DALYs) worldwide [5]. Eighty-five per cent of these impacts occurred in South Asia [5]; hence our focus on Bangladesh, India and Pakistan.

**Figure:**
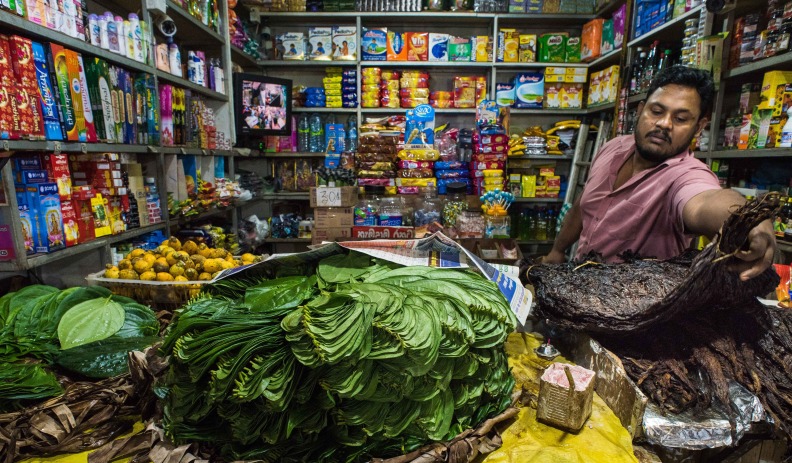
Photo: Smokeless tobacco for sale in a market in South Asia (with thanks to Ken Readshaw, for the use of the photograph).

All three of our target countries have signed up to the World Health Organization’s Framework Convention on Tobacco Control (WHO-FCTC) [6]. This sets out tobacco control measures, meant to be implemented by signatory WHO member countries, and designed to protect public health from the harms of tobacco. Such measures aim to reduce tobacco demand and supply, and include regulating the contents, packaging and labelling of tobacco products, warning people about the dangers of tobacco and banning tobacco sales to children. While this Framework has helped greatly in reducing smoking (especially in high-income countries), little focus has been applied to ST. This is because the harms of ST have historically been overshadowed by the even greater harmfulness of smoked cigarettes. Also, the diverse nature and cultural embeddedness of ST have rendered it a seemingly intractable problem. Furthermore, the informal nature of its supply chain makes ST a regulatory challenge. Consequently, ST control policies are poorly developed, badly implemented and rarely enforced. In general, they have not been supported by high-quality research, especially in low- and middle-income countries (LMICs) [7,8].

## ASTRA’S AIMS

The aims of ASTRA are to:

- gather evidence about how the policies recommended by the WHO-FCTC are being developed and implemented for ST in LMICs. Any gaps in ST control policies can then be addressed, through informed discussion with policy-makers. There will be a focus on young people, since the vast majority of ST users start their dependency during adolescence.
- develop and test the feasibility of interventions, such as behavioural support and/or medication, to help adult ST users to quit.

To address the above aims, ASTRA’s international teams are undertaking five inter-linked research studies, as follows.

The ASTRA-Youth study will assess the impact of WHO-FCTC policies on the uptake of ST among adolescents in Bangladesh, India and Pakistan. To do this, we will conduct school-based longitudinal surveys of adolescents (aged between 13 and 17 years), in two administrative areas in each country. Data will be gathered about teenagers’ ST habits, knowledge, awareness and attitudes towards smoking and ST. Data on exposure to tobacco, tobacco advertising and tobacco packaging and labelling will also be collected, and used to analyse local implementation of FCTC policies.

The ASTRA-Sellers study will assess the extent to which ST products are marketed and sold in violation of FCTC policies in Bangladesh, India and Pakistan. We will also identify barriers and facilitators to the effective implementation and enforcement of the relevant policies within the ST supply chain. Building on work that has already been carried out by ASTRA team members in the UK and South Asia [7,9], we will survey local shops that sell ST, and interview shopkeepers and their suppliers about how FCTC policies affect their business.

The ASTRA-Cessation study aims to optimise innovative behavioural and pharmacological interventions (such as nicotine replacement therapy) to support adults in Bangladesh, India and Pakistan who wish to quit using ST. A previously-tested successful behavioural support package for ST cessation [10] will be adapted for use in local contexts. Using a randomised trial design, we will test the interventions for feasibility and cultural acceptability, and assess whether further full-scale trials to estimate effectiveness and cost-effectiveness should be carried out.

The ASTRA-Economics study will estimate the economic impact of ST use at current consumption rates in Bangladesh and Pakistan, and estimate the impact of possible new strategies that could mitigate these costs.

The ASTRA-Policy study will develop a framework to measure ST control policies in South Asia.

## CAPACITY BUILDING AND SUSTAINABILITY

Further key features of ASTRA are capacity building and sustainability. With the help of policy makers (eg, WHO), advocates (eg, The UNION), funders and our stakeholders, we aim to support governments in making appropriate evidence-based and context-specific amendments to existing policies and regulations.

We will also build on our existing efforts to enhance capacity in applied health services research in South Asia. We plan to recruit a cohort of six Post-Doctoral Fellows (one based in the UK, one in Bangladesh and two each in India and Pakistan). They will be offered mentorship and a bespoke training programme to become independent researchers. We will use this capacity to support wider tobacco control efforts in the South Asia region.

ASTRA is an energetic group, with ambitious plans. We intend to seek future funding and forge further new collaborations, to exploit the opportunities offered by our research. We aim to expand our engagement with other academic, government and NGO (Non-Governmental Organization) partners. Steps are already under way to incorporate additional individuals and organizations with similar aims, as Affiliate Members of ASTRA.

## STAKEHOLDER ENGAGEMENT

Input from stakeholders is a key part of ASTRA, and our stakeholders’ opinions and advice will be crucial in informing our research studies and disseminating our results. We will also depend on our stakeholders for the sustainability and legacy of our work. Annual stakeholder workshop events have been held in each of our three partner countries, giving an opportunity for medical professionals, local experts in tobacco control, academics, policy-makers and advocates to have their say in tailoring our research programme for maximum benefit.

## THE IMPACT OF ASTRA

We aim for ASTRA to have a wide-reaching and long-lasting impact in the sphere of ST control. As described above, ASTRA’s international studies will further our knowledge about the supply chains, patterns of use and regulation of ST, thereby providing scientific evidence on which to base policy recommendations. Through our capacity building work, we will motivate and empower researchers working on reducing ST-related burden in our target countries.

The design of ASTRA is such that our collaborations will continue to evolve, even beyond the end of the programme. In the long run, ASTRA’s outputs could be used as a benchmark to evaluate the effectiveness and progress of ST policies in the region.

The UK is a member of the OECD (Organization for Economic Co-operation and Development), whose Development Assistance Committee (DAC) aims to provide aid (Official Development Assistance, ODA) to developing countries, to help them engage with the global economy and overcome poverty. Our target countries are on the ‘DAC-list’ of ODA recipients and hence ASTRA’s objectives are aligned with those of the OECD. ASTRA’s plans also fit with the United Nations’ Sustainable Development Goals (SDG), especially SDG 3 (promoting health and well-being) and SDG 10 (reducing inequalities).

We do not intend to lose sight of the fact that the ultimate beneficiaries of our programme will be the inhabitants of Bangladesh, India and Pakistan, where the combined populations of more than 1.7 billion people are currently exposed to a poorly-regulated ST market and all its associated harms. Going forward, ASTRA’s findings will also guide the implementation of strategies to reduce ST burden in other parts of the world.

ASTRA runs from 1 April 2018 until 31 March 2021. For a full list of ASTRA’s members, please see our website: https://www.york.ac.uk/healthsciences/research/public-health/projects/astra/.
